# Equilibrium and Kinetic Investigations of the Interaction of Model Platinum(II) Complex with DNA Constituents in Reference to the Antitumour Activity: Complex-Formation Reactions of [Pd(N,N-diethylethylenediamine)(H_2_O)_2_]^2+^ with Ligands of Biological Significance and Displacement Reactions of DNA Constituents

**DOI:** 10.1155/2009/512938

**Published:** 2009-08-17

**Authors:** Eman Mohamed Shoukry

**Affiliations:** Department of Chemistry, Faculty of Science, Taif University, 21944 Taif, Saudi Arabia

## Abstract

The [Pd(DEEN)Cl_2_] and [Pt(DEEN)Cl_2_] complexes were synthesized and characterized where DEEN = N,N-diethylethylenediamine. The stoichiometry and stability of the complexes formed between various biologically relevant ligands (amino acids, peptides, DNA constituents and dicarboxylic acids) and [Pd(DEEN)(H_2_O)_2_]^2+^ were investigated at 37°C and 0.16 M ionic strength. The stability constant of the complexes formed in solution were determined and the binding centres of the ligands were assigned. The concentration distribution diagrams of the complexes were evaluated The equilibrium constants for the displacement of representative coordinated ligands such as inosine, glycine or methionine by cysteine were calculated and the concentration distribution diagrams of the various species were evaluated. The kinetics of base hydrolysis of free and coordinated S-methylcysteine methyl ester was investigated. The mechanism of hydrolysis was discussed.

## 1. Introduction

Cis-platin [cis-diamminedichloroplatinum(II)] is one of the most active antitumour agents in clinical use [1]. However, it has a narrow spectrum of activity, and its clinical use is limited by undesirable side effects, including nephrotoxicity, ototoxicity, neurotoxicity, nausea, vomiting, and myelosuppression [2, 3]. In the search for new platinum anticancer drugs, great efforts are devoted to the design of complexes more efficient and less toxic than the reference drugs already in clinical use. For this purpose, the rational design of complexes and the study of relevant structure-activity relationships have been extended to families of new compounds having high structural diversity.

Pd(II) and Pt(II) amine complexes have the same structure, with a five orders of magnitude higher reactivity in the case of Pd(II) complexes, but similar thermodynamic parameters. Pd(II) complexes are good models for the analogous Pt(II) complexes in solution. Recent work in our laboratories focused on the equilibria of complex-formation reactions of cis-(diamine)palladium(II) complexes with DNA, the major target in chemotherapy of tumours, and biorelevant ligands as amino acids, peptides, dicarboxylic acids, and esters [4–10]. In the present project we have synthesised and characterised the [Pt(DEEN)Cl_2_] and [Pd(DEEN)Cl_2_] complexes. The thermodynamic behaviour of the Pd(II) complex was investigated. The amine investigated has two ethyl groups attached to one nitrogen atom of ethylenediamine. The two ethyl groups will create steric hinderence with the incoming ligand as DNA. This will tune the reactivity of the complex to be similar to the antitumour platinum-amine complex. The equilibrium studies are conducted at 37°C and ionic strength 0.16 M. This condition is similar to what is exist in biological fluids. The sulphur ligands as cysteine or glutathione have high affinity for Pd(II) and Pt(II) complexes. These ligands will compete with the DNA for the reaction with any antitumour agent. Therefore, it is of biological significance to evaluate the equilibrium constants for the displacement reaction of model ligands as inosine, glycine, or methionine by cysteine. These equilibrium constants may give a measure of the effectiveness of the antitumour agent.

## 2. Experimental

### 2.1. Materials

 K_2_PdCl_4_, K_2_PtCl_4_, N,N-diethylethylendiamine, and cyclobutanedicarboxylic acid were obtained from Aldrich. The amino acids and related compounds (glycine, alanine, *β*-alanine, *γ*-aminobuteric acid, *β*-phenylalanine, valine, proline, hydroxyproline, iso-leucine, ethanolamine · HCl, serine, histidine, histamine dihydro-chloride, ornithine, lysine, cysteine, methionine, threonine, S-methylcysteine, aspartic acid glutamic acid, and methylamine · HCl) were provided by Sigma Chemical Co. The peptides used (glycinamide, glycylglycine, glycylleucine, asparagines, and glutamine) and the dibasic acids used (cyclobutane dicarboxylic acid, malonic acid, oxalic acid, succinic acid, adipic acid, and fumaric acid) were all provided by BDH-Biochemicals Ltd, Poole, UK. The DNA constituents (inosine, inosine 5′-monophosphate, adenine, guanosine, guanosine 5′-monophosphate, adenosine, cytosine, thymidine, cytidine 5′-monophosphate, uracil, and uridine 5′-monophosphate) were provided by Sigma Chemical Co. S-methylcysteine methyl ester was obtained from Sigma Chem. Co. For equilibrium studies, [Pd(DEEN)Cl_2_] was converted into the diaqua complex by treating it with two equivalents of AgNO_3_ as described before [11]. The ligands in the form of hydrochlorides were converted into the corresponding hydronitrates. Cytosine, guanosine, and the nucleotides were prepared in the protonated form with standard HNO_3_ solution. All solutions were prepared in deionized water.

### 2.2. Synthesis

[Pd(DEEN)Cl_2_] was prepared by dissolving K_2_PdCl_4_ (2.82 mmol) in 10 mL water with stirring. The clear solution of [PdCl_4_]^2−^ was filtered, and N,N-diethylethylenediamine (2.82 mmol), dissolved in 10 mL H_2_O, was added dropwise to the stirred solution. The pH was adjusted to 2-3 by the addition of HCl and/or NaOH. A yellowish-brown precipitate of [Pd(DEEN)Cl_2_] was formed and stirred for a further 30 minute at 50°C. After filtering off the precipitate, it was thoroughly washed with H_2_O, ethanol, and diethylether. A yellow powder was obtained. Anal. Calcd. for C_6_H_16_N_2_PdCl_2_: C, 24.54; H, 5.45; N, 9.54. Found: C, 24.51; H, 5.46; N, 9.44. The IR spectrum of Pd(DEEN)Cl_2_ exhibits strong NH absorption band in the range 3113-3207 cm^−1^. *δ* (NH) bands are observed at 1580–1609 cm^−1^. The Pd-N absorption was detected at 439 cm^−1^. The chloro complex was converted into the corresponding aqua complex in solution by addition of two equivalents of AgNO_3_, heating to 40–50°C for 3 hours, and removing the precipitated AgCl by filteration.

[Pt(DEEN)Cl_2_] was prepared by dissolving K_2_PtCl_4_ (2 mmol) in 10 mL water with stirring. The clear solution of [PtCl_4_]^2−^ was filtered, and N,N-diethylethylenediamine (2 mmol), dissolved in 10 mL water, was added dropwise to the stirred solution. The solution mixture was refluxed at 70°C for 6 hours. The solution is evaporated to 5 mL. A yellow precipitate of [Pt(DEEN)Cl_2_] is formed. The precipitate is filtered, thoroughly washed with water, ethanol, and diethylether. Anal. Calcd for C_6_H_16_N_2_PtCl_2_ · H_2_O: C, 17.99; H,4.50; N, 7.00. Found: C, 18.69; H,4.24; N, 6.95%. The IR spectrum of [Pt(DEEN)Cl_2_.H_2_O] exhibits strong bands (H_2_O) in the range 3479-3544 cm^−1^, strong NH absorption band in the range 3128–3239 cm^−1^. *δ* (NH) band is observed at 1608 cm^−1^. The Pt-N absorption was detected at 441 cm^−1^.

### 2.3. Apparatus

Potentiometric titrations were performed with a Metrohm 686 titroprocessor equipped with a 665 Dosimat. The titroprocessor and electrode were calibrated with standard buffer solutions, prepared according to NBS specification [12]. All titrations were carried out at 37.0 ± 0.1°C in purified nitrogen atmosphere using a titration vessel described previously [13]. IR spectra were measured on a 8001-PC FT-IR Shimadzu spectrophotometer using KBr pellets.

### 2.4. Procedure and Measuring Technique

The acid dissociation constants of the ligands were determined by titrating 1.00 mmol samples of each with standard NaOH solutions. Ligands were converted into their protonated form with standard HNO_3_ solutions. The acid dissociation constants of the coordinated water molecules in [Pd(DEEN)(H_2_O)_2_]^2+^ were determined by titrating 1.00 mmol of complex with standard 0.05 M NaOH solution. The formation constants of the complexes were determined by titrating solution mixtures of [Pd(DEEN)(H_2_O)_2_]^2+^ (1.00 mmol) and the ligand in the concentration ratio of 1 : 1 for amino acids, peptides, and dicarboxylic acids and in the ratio of 1 : 2 (Pd:ligand) for the DNA constituents. The titrated solution mixtures each had a volume of 40 mL, and the titrations were carried out at 37°C and 0.1 M ionic strength (adjusted with NaNO_3_). A standard 0.05 M NaOH solution was used as titrant. The pH metre readings were converted to hydrogen ion concentration by titrating a standard HNO_3_ solution (0.01 M), the ionic strength of which was adjusted to 0.1 M with NaNO_3_, with standard NaOH (0.05 M) at 37°C. The pH was plotted against p[H]. The relationship pH-p[H] = 0.05 was observed. 

The species formed were characterized by the general equilibrium

(1) $$\text{pM}+\text{qL}+\text{rH}⇌{\left(\text{M}\right)}_{\text{p}}{\left(\text{L}\right)}_{\text{q}}{\left(\text{H}\right)}_{\text{r}},$$

for which the formation constants are given by

(2) $$\beta_{\text{pqr}}=\frac{\left[{\left(\text{M}\right)}_{\text{p}}{\left(\text{L}\right)}_{\text{q}}{\left(\text{H}\right)}_{\text{r}}\right]\;}{{\left[\text{M}\right]}^{\text{p}}{\left[\text{L}\right]}^{\text{q}}{\left[\text{H}\right]}^{\text{r}}},$$

where M, L and H stand for [Pd(DEEN)(H_2_O)_2_]^2+^ ion, ligand, and proton, respectively. The calculations were performed using the computer program [14] MINIQUAD-75. The stoichiometry and stability constants of the complexes formed were determined by trying various possible composition models for the systems studied. The model selected was that which gave the best statistical fit and was chemically consistent with the magnitudes of various residuals, as described elsewhere [14]. Tables 1  and 2  list the stability constants together with their standard deviations and the sum of the squares of the residuals derived from the MINIQUAD output. The concentration distribution diagrams were obtained with the program SPECIES [15] under the experimental condition used. 

 The hydrolysis kinetics of the complex ester was monitored by pH-stat technique, [13, 16, 17] by using the titroprocessor operating in the SET mode. The hydrolysis was investigated using an aqueous solution (40 mL) containing a mixture of [Pd(DEEN)(H_2_O)_2_]^2+^ (1 mmol) and S-methylcysteine methyl ester (1 mmol), and the ionic strength was adjusted to 0.16 M with NaNO_3_. The pH of the mixture was progressively raised to the desired value. The reaction was monitored by addition of NaOH solution to maintain the given pH. The data fitting was performed with the OLIS KINFIT set of programs [18] as described previously [19, 20].

## 3. Results and Discussion

The acid dissociation constants of the ligands were determined under the experimental conditions 37°C and constant 0.16 M ionic strength (adjusted with NaNO_3_), which were also used for determining the stability constants of the Pd(II) complexes.

### 3.1. Hydrolysis of [Pd(DEEN) (H_2_O)_2_]^2+^

The [Pd(DEEN)(H_2_O)_2_]^2+^ ion may undergo hydrolysis. Its acid-base chemistry was characterized by fitting the potentiometric data to various acid-base models. The best-fit model was found to be consistent with the formation of three species: 10-1, 10-2, and 20-2, as given in reactions (3)–(5). Trials were made to fit the potentiometric data assuming the formation of the monohydroxo-bridged dimer, 20-1, but this resulted in a very poor fit to the data. The dimeric species 20-2 was detected by Nagy and Sóvágó [21], for a similar system:

(3) $$\underset{100}{{\left[\text{Pd}\left(\text{DEEN}\right){\left({\text{H}}_{\text{2}}\text{O}\right)}_{\text{2}}\right]}^{\text{2+}}}\overset{{\text{K}}_{\text{a}1}}{⇋}\underset{10\text{-}1}{{\left[\text{Pd}\left(\text{DEEN}\right)\left({\text{H}}_{\text{2}}\text{O}\right)\left(\text{OH}\right)\right]}^{\text{+}}}+{\text{H}}^{+},$$

(4) $$\underset{10\text{-}1}{{\left[\text{Pd}\left(\text{DEEN}\right)\left({\text{H}}_{\text{2}}\text{O}\right)\left(\text{OH}\right)\right]}^{\text{+}}}\overset{{\text{K}}_{\text{a}2}}{⇋}\underset{10\text{-}2}{\left[\text{Pd}\left(\text{DEEN}\right){\left(\text{OH}\right)}_{2}\right]+{\text{H}}^{+}},$$

(5) $$\underset{10\text{-}1}{{2\left[\text{Pd}\left(\text{DEEN}\right)\left({\text{H}}_{\text{2}}\text{O}\right)\left(\text{OH}\right)\right]}^{\text{+}}}\overset{{\text{K}}_{\text{dimer}}}{⇋}\underset{20\text{-}2}{{\left[\text{Pd}\left(\text{DEEN}\right){\left(\text{OH}\right)}_{2}\text{Pd}\left(\text{DEEN}\right)\right]}^{2+}}.$$

The pK_a1_ and pK_a2_ values for [Pd(DEEN)(H_2_O)_2_]^2+^ are 5.11 and 9.69, respectively, The equilibrium constant for the dimerization reaction (5) can be calculated by (6) and amounts to 3.02:

(6) $$\log\;\;{\text{K}}_{\text{dimer}}=\log\;\;\beta_{20\text{-}2}-2\log\;\;\beta_{10\text{-}1}.$$

**Figure 1:**
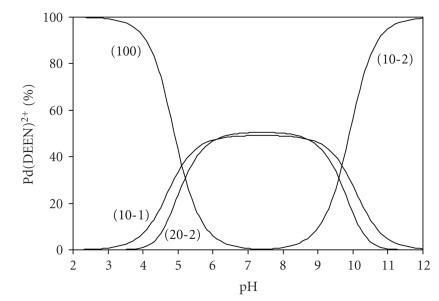
Concentration distribution diagram for the hydrolysis of [Pd(DEEN)(H_2_O)_2_]^2+^.

The distribution diagram for [Pd(DEEN)(H_2_O)_2_]^2+^ and its hydrolyzed species is given in Figure 1 and reveals that the concentration of the monohydroxo species 10-1, and the dimeric species 20-2 increase with increasing pH, predominate in the pH range 6–8, and reach a maximum concentration of ~50%. A further increase in pH is accompanied by an increase in the dihydroxo species (10-2), which is the main species above pH ~10.0. This reveals that, in the physiological pH range, that is, at pH 6-7, the monohydroxo complex (10-1) predominates and can interact with the DNA subunits. At higher pH the inert dihydroxo complex will be the major species, and consequently the ability of DNA to bind the Pd(amine) complex will decrease significantly.

### 3.2. Amino Acid Complexes

Analysis of the titration data for the Pd(DEEN)-amino acid system showed the formation of 1 : 1 species. Histidine, ornithine, lysine, glutamic acid, aspartic acid, and cysteine form, in addition to 1 : 1 complexes, the monoprotonated species. The pk_a_ of the protonated complex was calculated from (7):

(7) $$\text{p}{\text{k}}_{\text{a}}=\log\;\;\beta_{111}-\log\;\;\beta_{110}$$

The pk_a_ values of the protonated species are 2.95 for histidine, 7.20 for ornithine, 8.58 for lysine, 3.17 for glutamic acid and, 3.39 for aspartic acid. The stability constants of the histidine, ornithine, and lysine complexes are higher than those of simple amino acids. This indicates that these amino acids coordinate via the two nitrogen centres, that is, imidazole and amino groups in the case of histidine, and by two amino groups in the case of ornithine and lysine. This is in line with the strong affinity of Pd(II) for nitrogen donor centres. Aspartic acid and glutamic acid have two carboxylic and one amino groups as potential binding centres. They may coordinate either via the two carboxylate groups or by the amino group and one carboxylate group. The stability constants of their complexes are in the range of those of amino acids. This may reveal that aspartic acid and glutamic acid coordinate via the amino and one carboxylate groups. Serine and threonine have an extra binding centre on the *β*-alcoholate group. This group was reported [22] to participate in metal complex formation. The potentiometric data is much better fitted assuming the formation of the complex species 110 and 11-1. This reveals that the *β*-alcoholate group participates in complex formation through induced ionization of the alcoholic group forming the species 11-1. The pk^H^ value of the *β*-alcoholate incorporated in the Pd(II) complex (log *β*_110_ − log *β*_11-1_) is 7.97 and 7.88 for serine and threonine, respectively. Also, ethanolamine forms the complex species 110, 120, and 11-1, and the log *β*_110_ value for ethanolamine complex is smaller than that for amino acids. This may be due to the coordination of ethanolamine at low pH occurring through the amino and neutral alcohol groups, while in the case of serine and threonine the coordination is through amino and carboxylate groups. At high pH, the hydroxyl group is coordinated and undergoes induced ionization forming the species 11-1. The pk^H^ value of the coordinated alcohol group in ethanolamine (4.98) is smaller than that of serine and threonine. This is consistent with the reaction scheme where the alcohol group in ethanolamine is coordinated with the Pd(DEEN)^2+^, while the alcohol group in serine and threonine is competing with the carboxylate group in binding to Pd(DEEN)^2+^ ion. Due to coordination of the alcohol group by donation of the electron pair on the oxygen to the metal centre, the OH bond is considerably weakened, and thus the ionization of a proton occurs at a lower pH. 

**Figure 2:**
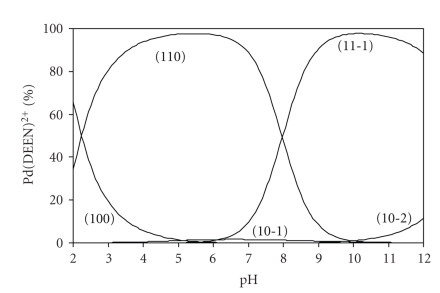
Concentration distribution diagram for various complex species in Pd(DEEN)^2+^-serine system.

The distribution diagram for the serine complex, given in Figure 2, shows that the complex species with coefficients 110 reaches the maximum degree of formation (~97%) at pH 5.0 to 6.0, that is, in the physiological pH range. However, the species 11-1 predominates after pH 8.5 and attains the maximum concentration of ~98% at pH ~9.5.

### 3.3. Peptide Complexes

The potentiometric data for the peptide complexes were fitted on the basis of formation of the complexes with stoichiometric coefficients 110 and 11-1 according to the following equilibria, where HL is peptide:

(8) $$\begin{array}{ccc} \underset{100}{{\left[\text{Pd}\left(\text{DEEN}\right){\left({\text{H}}_{\text{2}}\text{O}\right)}_{\text{2}}\right]}^{\text{2+}}\text{+}{\text{L}}^{-}} & \overset{\text{K}}{⇋} & \underset{110}{{\left[\text{Pd}\left(\text{DEEN}\right)\text{L}\right]}^{\text{+}}\text{ + 2}{\text{H}}_{\text{2}}\text{O}},\\ \underset{110}{{\left[\text{Pd}\left(\text{DEEN}\right)\text{L}\right]}^{\text{+}}} & \overset{{\text{K}}^{\text{H}}}{⇋} & \underset{11\text{-}1}{{\left[\text{Pd}\left(\text{DEEN}\right)\text{L}{\text{H}}_{-\text{1}}\right]}^{}+{\text{H}}^{+}}. \end{array}$$

The 110 complex is formed via coordination of the amine and carbonyl groups. On increasing the pH, the coordination site should switch from the carbonyl oxygen to the amide nitrogen with release of the amide hydrogen forming the complex [Pd(DEEN)(LH_−1_)]. Such changes in coordination centres are now well documented [23]. The pK^H^ values of the amide groups incorporated in the Pd(II) complexes (log *β*_110_ − log *β*_11-1_) are in the 3.65–10.80 range. It is noteworthy that the pK^H^ for glycinamide complex is lower than that of other peptides. This signifies that the more bulky substituent group on the peptide may serve to hinder the structural change in going from the protonated to the deprotonated complexes. The pK^H^ of the glutamine complex is markedly higher than that for other peptide complexes. This is ascribed to the formation of a seven-membered chelate ring, which would probably be more strained and therefore less favoured. 

**Figure 3:**
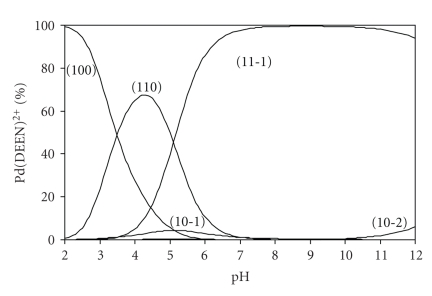
Concentration distribution diagram for various complex species in Pd(DEEN)^2+^ -glycylglycine system.

The relative magnitude of the pK^H^ values of the Pd(II) complexes with peptides has interesting biological implications. Under normal physiological conditions (pH 6-7) the peptide would coordinate to [Pd(DEEN)(H_2_O)_2_]^2+^ in entirely different fashions. Glutaminate would exist solely in the protonated form, whereas the other peptides would be present entirely in the deprotonated form. In addition, the slight difference in the side chain of the peptides produces dramatic differences in their behaviour towards the palladium species. The speciation diagram of glycylglycine complex is given in Figure 3. The Pd(DEEN)(L)^+^ (110) species starts to form at pH 2.0 and with increasing of pH, its concentration increases reaching the maximum of 70% at pH 4.3. Further increase of pH is accompanied by a decrease in Pd(DEEN)(L)^+^ complex concentration and an increase of Pd(DEEN)(LH_−1_) complex formation.

### 3.4. Dicarboxylic Acid Complexes

In the Pd(DEEN)-dicarboxylic acid system, the results showed the presence of the 1 : 1 species and its protonated form. The results in Table 2 show that the adipic acid complex is the least stable as the complex involves the formation of the least stable eight-membered chelate ring. The pK_a_ values of the protonated species for [Pd(DEEN)HL]^+^ are in the range 1.65 – 4.95. These values are lower than those for the HL^−^ ion see Table 2. The lowering of the pK_a_ is due to acidification of the second carboxylic acid group upon coordination of Pd(II) to one carboxylate group [24]. 

**Figure 4:**
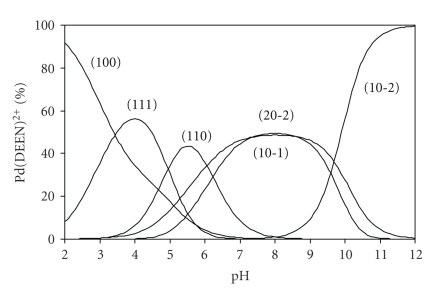
Concentration distribution diagram for various complex species in Pd(DEEN)^2+^-succinic acid System.

From the concentration distribution diagram of the succinic acid complex, given in Figure 4, it is interesting to note that the monoprotonated species attains its maximum concentration of 58% at pH 3.8. This form has one coordination site available for binding to DNA. Such species was documented to be the active form in the case of carboplatin [25].

### 3.5. DNA Complexes

DNA constituents, such as adenosine, cytosine, uracil, and thymidine, form 1 : 1 and 1 : 2 complexes with the Pd(DEEN)^2+^ ion. However, inosine, adenine and nucleotides such as inosine-5′-monophosphate, guanosine-5′-monophosphate, cytidine-5′-monophosphate and uridine-5′-monophosphate, form the monoprotonated complex, in addition to the formation of 1 : 1 and 1 : 2 complexes. The pK_a_ value of the protonated inosine complex is 4.51. This value corresponds to N_1_H. The lowering of this value with respect to that of free inosine (pK_a_ = 8.43) is due to acidification upon complex formation [26]. The pK_a_ values of the protonated nucleotide complexes are 5.75, 6.03, 5.07, and 6.01 for inosine-5′-monophosphate, guanosine-5′-monophosphate, cytidine-5′-monophosphate, and uridine-5′-monophosphate respectively. 

The pyrimidines uracil, uridine-5′-monophosphate, thymidine-5^,^-monophosphate, and thymidine have basic nitrogen donor atoms (N_3_)^26^ as a result of the high pK_a_ values of pyrimidines (pK_a_ > 9), and the complexes formed predominates above pH 8.5. Both cytosine and cytidine-5′-monophosphate undergo N_3_^27^ protonation under mild acidic conditions. The values obtained for their protonation constants are 4.45 and 6.12, respectively. The lower values of the stability constants of their complexes, Table 2, reflect the difference in the basicity of the donor site. 

It was shown above that N-donor ligands such as DNA constituents have affinity for [Pd(DEEN)(H_2_O)_2_]^2+^, which may have important biological implications since the interaction with DNA is thought to be responsible for the antitumour activity of related complexes. However, the preference of Pd(II) to coordinate to S-donor ligands was demonstrated as shown in Tables 1  and 2. These results suggest that Pd(II)-N adducts can easily be converted into Pd-S adducts [27]. Consequently, the equilibrium constant for such conversion is of biological significance. Consider inosine as a typical DNA constituent (presented by HA) and cysteine as a typical thiol ligand (presented by H_2_B). The equilibria involved in complex-formation and displacement reactions are

(9) $$\begin{array}{c} \text{HA}⇌{\text{H}}^{+}+{\text{A}}^{-},\\ {\text{H}}_{2}\text{B}⇌2{\text{H}}^{+}+{\text{B}}^{2-},\\ \begin{array}{ccc} {\left[\text{Pd}\left(\text{DEEN}\right){\left({\text{H}}_{2}\text{O}\right)}_{2}\right]}^{2+}+{\text{A}}^{-} & ⇌ & {\left[\text{Pd}\left(\text{DEEN}\right)\text{A}\right]}^{+}+2{\text{H}}_{2}\text{O}\\ \left(100\right) & & \left(110\right) \end{array}, \end{array}$$

(10) $$\begin{array}{c} \begin{array}{cc} \beta_{110}^{\left[\text{Pd}\left(\text{DEEN}\right)\text{A}\right]+} & =\frac{\left[\text{Pd}\;\left(\text{DEEN}\right){\text{A}}^{+}\right]}{{\left[\text{Pd}\left(\text{DEEN}\right){\left({\text{H}}_{2}\text{O}\right)}_{2^{}}\right]}^{2+}\left[{\text{A}}^{-}\right]}, \end{array} \end{array}$$

(11) $$\begin{array}{c} \begin{array}{ccc} {\left[\text{Pd}\left(\text{DEEN}\right){\left({\text{H}}_{2}\text{O}\right)}_{2}\right]}^{2+}+{\text{B}}^{2-} & ⇌ & \left[\text{Pd}\left(\text{DEEN}\right)\text{B}\right]+2{\text{H}}_{2}\text{O}\\ 100 & & 110 \end{array}, \end{array}\;$$

(12) $$\begin{array}{c} \begin{array}{cc} \beta_{110}^{\left[\text{Pd}\left(\text{DEEN}\right)\text{B}\right]} & =\frac{\left[\text{Pd}\left(\text{DEEN}\right)\text{B}\right]}{{\left[\text{Pd}\left(\text{DEEN}\right){\left({\text{H}}_{2}\text{O}\right)}_{2^{}}\right]}^{2+}\left[{\text{B}}^{2-}\right]} \end{array} \end{array}$$

(13) $$\begin{array}{c} \begin{array}{ccc} {\left[\text{Pd}\left(\text{DEEN}\right)\left(\text{A}\right)\right]}^{+}+{\text{B}}^{2-} & \overset{{\text{K}}_{\text{eq}}}{⇋} & \left[\text{Pd}\left(\text{DEEN}\right)\left(\text{B}\right)\right]+{\text{A}}^{-} \end{array} \end{array}$$

The equilibrium constant for the displacement reaction given in (13) is given by

(14) $${\text{K}}_{\text{eq}}=\frac{\left[\text{Pd}\left(\text{DEEN}\right)\left(\text{B}\right)\right]\left[\;{\text{A}}^{-}\right]}{\left[\text{Pd}\left(\text{DEEN}\right){\left(\text{A}\right)}^{+}\right]\left[{\text{B}}^{2-}\right]}.$$

Substitution from (10) and (12) in (14) results in:

(15) $${\text{K}}_{\text{eq}}=\frac{\beta_{110}^{\left[\text{Pd}\left(\text{DEEN}\right)\text{B}\right]}}{\beta_{110}^{{\left[\text{Pd}\left(\text{DEEN}\right)\text{A}\right]}^{+}}}$$

where log *β*_110_ values for [Pd(DEEN)(A)]^+^ and [Pd(DEEN)B] complexes taken from Table 2 amount to 7.38 and 14.11, respectively, and by substitution in (15) results in log K_eq_ = 6.73. In the same way the equilibrium constants for the displacement of coordinated inosine by glycine and methionine are log K_eq_ = 2.95 and 1.94, respectively. These values clearly indicate how sulfhydryl ligands such as cysteine and, by analogy, glutathione are effective in displacing the DNA constituent, that is, the main target in tumour chemotherapy. Chelated cyclobutanedicarboxylate may undergo displacement reaction with inosine. log K_eq_ for such a reaction was calculated as described above and amounts to 1.27. The low value of the equilibrium constant for the displacement reaction of coordinated cyclobutanedicarboxylate by inosine is of biological significance since it is in line with the finding that carboplatin interacts with DNA through ring opening of chelated CBDCA and not through displacement of CBDCA.

### 3.6. Kinetics of Hydrolysis of Amino Acid Esters

**Scheme 1:**
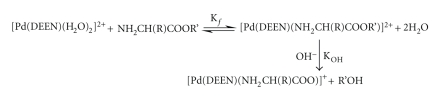


The hydrolysis of amino acid esters can be presented as shown in Scheme 1. 

The equilibrium constant for S-methylcysteine methyl ester (K_f_) is sufficiently large that coordination of the ester is readily accomplished. The kinetic data, name, the volume of base added to keep the pH constant versus time, could be fitted by a single exponential function. A plot of k_obs_ versus hydroxide ion concentration is linear and follows the rate expression k_obs_ = k_o_ + k_OH_[OH^−^], where k_o_ represents the rate constant for the water-catalyzed pathway and k_OH_ the rate constant for the base-catalyzed pathway, the kinetic data are given in Table 3. The linear dependence of the rate constant on the OH^−^ concentration is consistent with direct attack of OH^−^ ion on the ester carbonyl group. The catalysis ratio C = k_OH_/k_OH^ester^_, where k_OH^ester^_ represents the rate constant for the hydrolysis of the free amino acid ester, is given in Table 4. The catalysis ratio for coordinated cysteine methyl ester equals 91.0. A catalysis ratio of such low value is consistent with the structural formula for the mixed-ligand complex, in which there is no direct interaction between the Pd^II^ ion and the ester carbonyl group. The relatively low catalysis ratio is probably due to activation by induction through the coordinated nitrogen atom as reported previously [28, 29]. 

## Figures and Tables

**Scheme 2:**
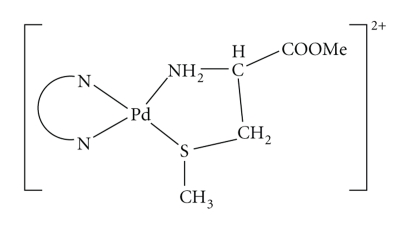


**Table 1:** Formation constants for complexes of [Pd(DEEN)(H_2_O)_2_ ]^2+^ with amino acids at 37°C and 0.16 M ionic strength.

Ligand	MLH^a^	log *β* ^b^	Ligand	M L H^a^	log *β* ^b^
OH^−^	1 0 − 1	−5.11(0.01)	Glycine	0 1 1	9.20(0.02)
	1 0 − 2	−14.80(0.02)		0 1 2	11.20(0.03)
	2 0 − 2	−7.20(0.04)		1 1 0	10.33(0.02)
Alanine	0 1 1	9.27(0.01)	*β*-Phenylalanine	0 1 1	9.12(0.01)
	0 1 2	12.17(0.02)		0 1 2	11.01(0.03)
	1 1 0	10.16(0.02)		1 1 0	9.86(0.02)
*γ*-Aminobutyric acid	0 1 1	9.63(0.00)	*β*-Alanine	0 1 1	9.70(0.02)
	0 1 2	13.10(0.02)		0 1 2	13.20(0.03)
	1 1 0	7.61(0.02)		1 1 0	9.50(0.02)
Valine	0 1 1	9.01(0.01)	Proline	0 1 1	10.06(0.01)
	0 1 2	11.48(0.02)		0 1 2	11.81(0.05)
	1 1 0	9.90(0.03)		1 1 0	10.51(0.10)
Iso-Leucine	0 1 1	9.46(0.01)	Histamine	0 1 1	9.34(0.01)
	0 1 2	11.74(0.02)		0 1 2	15.20(0.02)
	1 1 0	10.40(0.02)		1 1 0	12.85(0.08)
Histidine	0 1 1	8.84(0.01)	Ethanolamine	0 1 1	9.16(0.01)
	0 1 2	14.74(0.02)		1 1 0	6.99(0.02)
	0 1 3	16.81(0.06)		1 2 0	12.00(0.04)
	1 1 0	13.37(0.01)		1 1 -1	2.01(0.04)
	1 1 1	16.32(0.03)			(pK^H^ = 4.98)
Serine	0 1 1	8.59(0.01)	Threonine	0 1 1	8.79(0.01)
	0 1 2	10.95(0.02)		0 1 2	10.85(0.02)
	1 1 0	10.01(0.01)		1 1 0	9.98(0.07)
	1 1-1	2.04(0.02)		1 1-1	2.10(0.08)
		(pK^H^ = 7.97)			(pK^H^ = 7.88)
Ornithine	0 1 1	9.81(0.02)	Lysine	0 1 1	9.90(0.02)
	0 1 2	18.16(0.02)		0 1 2	18.80(0.03)
	0 1 3	20.01(0.03)		0 1 3	21.00(0.03)
	1 1 0	12.90(0.05)		1 1 0	10.12(0.05)
	1 1 1	20.10(0.04)		1 1 1	18.70(0.02)
Aspartic acid	0 1 1	9.31(0.02)	Glutamic acid	0 1 1	9.34(0.02)
	0 1 2	12.79(0.03)		0 1 2	13.15(0.03)
	0 1 3	14.50(0.03)		0 1 3	15.73(0.05)
	1 1 0	8.53(0.03)		1 1 0	8.70(0.03)
	1 1 1	11.92(0.04)		1 1 1	11.87(0.02)
S-methylcysteine methyl ester	0 1 1	8.51(0.02)	Methionine	0 1 1	8.76(0.02)
	0 1 2	10.40(0.02)		0 1 2	11.00(0.03)
	1 1 0	9.51(0.01)		1 1 0	9.32(0.04)
Hydroxyproline	0 1 1	9.20(0.01)	Methylamine	0 1 1	9.32(0.01)
	0 1 2	11.00(0.02)		1 1 0	7.11(0.06)
	1 1 0	9.91(0.03)		1 2 0	12.53(0.04)
Cysteine	0 1 1	10.00(0.01)			
	0 1 2	18.21(0.02)			
	0 1 3	19.62(0.02)			
	1 1 0	14.11(0.03)			
		18.20(0.04)			

^a^M, L, and H are the stoichiometric coefficients corresponding to Pd(DEEN), amino acid, and H^+^, respectively; the coefficient –1, refers to a proton loss. ^b^log *β* of Pd(DEEN)-amino acids. Standard deviations are given in parentheses; sum of square of residuals are less than 5E-7, pK^H^ = log *β*_110_ − log *β*_11-1_.

**Table 2:** Formation constants for complexes of [Pd(DEEN)(H_2_ O)_2_]^2+^ with peptides, dibasic acids, and DNA units at 37°C and 0.16 M ionic strength.

Ligand	M L H^a^	log *β* ^b^	Ligand	M L H^a^	log *β* ^b^
Glycinamide			Cyclobutane-1,1- dicarboxylic acid		
	0 1 1	7.50(0.02)		0 1 1	5.37(0.01)
	1 1 0	7.81(0.02)		0 1 2	8.17(0.01)
	1 1-1	4.16(0.01)		1 1 0	6.11(0.02)
		(pK^H^ = 3.65)		1 1 1	7.76(0.05)
Glycylglycine			Succinic acid		
	0 1 1	7.77(0.01)		0 1 1	5.24(0.02)
	0 1 2	10.81(0.01)		0 1 2	9.17(0.03)
	1 1 0	7.71(0.01)		1 1 0	4.21(0.00)
	1 1-1	2.61(0.08)		1 1 1	9.16(0.01)
Aspargine			Malonic acid		
	0 1 1	8.35(0.01)		0 1 1	5.01(0.02)
	0 1 2	10.51(0.03)		0 1 2	7.65(0.03)
	1 1 0	9.30(0.02)		1 1 0	5.68(0.02)
	1 1-1	−0.50(0.04)		1 1 1	8.15(0.06)
		(pK^H^ = 9.80)			
Glycylleucine			Adipic Acid		
	0 1 1	7.91(0.01)		0 1 1	5.25(0.03)
	1 1 0	7.35(0.03)		0 1 2	9.14(0.04)
	1 1-1	1.98(0.08)		1 1 0	3.98(0.00)
		(pK^H^ = 5.37)		1 1 1	8.26(0.00)
Glutamine			Oxalic acid		
	0 1 1	8.77(0.01)		0 1 1	3.76(0.02)
	0 1 2	10.78(0.02)		0 1 2	5.37(0.03)
	1 1 0	9.40(0.02)		1 1 0	5.84(0.07)
	1 1-1	−1.40(0.05)		1 1 1	8.06(0.07)
		(pK^H^ =10.80)			
Inosine			Fumaric acid		
	0 1 1	8.43(0.02)		0 1 1	4.27(0.04)
	1 1 0	7.38(0.03)		0 1 2	6.63(0.04)
	1 2 0	10.64(0.05)		1 1 0	4.23(0.04)
	1 1 1	11.89(0.05)		1 1 1	7.74(0.06)
Inosine-5′-monophosphate			Cytidine-5′-monophosphate		
	0 1 1	8.83(0.02)		0 1 1	6.12(0.02)
	0 1 2	15.07(0.03)		0 1 2	10.42(0.03)
	1 1 0	8.18(0.03)		1 1 0	5.59(0.07)
	1 2 0	13.35(0.04)		1 2 0	8.37(0.09)
	1 1 1	13.93(0.04)		1 1 1	10.66(0.05)
Adenine			Uridine-5′-monophosphate		
	0 1 1	9.29(0.03)		0 1 1	9.23(0.01)
	0 1 2	13.32(0.04)		0 1 2	15.12(0.02)
	1 1 0	9.17(0.11)		1 1 0	9.14(0.01)
	1 2 0	13.77(0.02)		1 2 0	13.82(0.02)
	1 1 1	18.36(0.02)		1 1 1	15.15(0.04)
Cytosine			Uracil		
	0 1 1	4.45(0.02)		0 1 1	8.98(0.01)
	1 1 0	5.68(0.03)		1 1 0	8.33(0.04)
	1 2 0	8.51(0.04)		1 2 0	13.70(0.08)
Guanosine			Guanosine-5′-monophosphate		
	0 1 1	8.91(0.01)		0 1 1	9.18(0.02)
	0 1 2	11.01(0.02)		0 1 2	15.12(0.03)
	1 1 0	10.18(0.06)		1 1 0	9.03(0.06)
	1 2 0	18.88(0.06)		1 2 0	13.44(0.19)
				1 1 1	15.06(0.01)
Adenosine			Thymidine		
	0 1 1	3.40(0.01)		0 1 1	9.28(0.04)
	1 1 0	2.74(0.04)		1 1 0	8.05(0.08)
	1 2 0	5.15(0.00)		1 2 0	13.22(0.04)

^*a*^M, L and H are the stoichiometric coefficients corresponding to Pd(DEEN), ligands,and H^+^, respectively; the coefficient –1 refers to a proton loss. ^b^log *β* of Pd(DEEN)- ligands. Standard deviations are given in parentheses; sum of square of residuals are less than 5*e* ^−7^ *, *pK^H^ = log   *β*_110_ − log   *β*_11-1_.

**Table 3:** Kinetic data for hydrolysis of [Pd(DEEN)(cysteine methyl ester)]^2+^ at 25°C and 0.1 M ionic strength.

pH	[OH^−^]/M	k_obs_/s^−1^
8.8	7.59E − 06	4.95E − 04
9.0	1.20E − 05	7.67E − 04
9.2	1.91E − 05	1.39E − 03
9.4	3.02E − 05	1.88E − 03
9.6	4.79E − 05	3.36E − 03

**Table 4:** Kinetic data for the hydrolysis of [Pd(DEEN)(cysteine methyl ester)]^2+^ at 25°C and 0.1 M ionic strength.

k_OH_	k_o_	k_OH^(ester)a^_	k_OH_/k_OH^(ester)^_
M^−1^s^−1^	s^−1^	M^−1^s^−1^	

6.98E + 01	9.270E − 07	0.767	9.10E + 01

^a^Data taken from [16, 17]
